# Evaluation of Primary DNA Damage in Young Healthy Females Based on Their Dietary Preferences

**DOI:** 10.3390/nu15092218

**Published:** 2023-05-08

**Authors:** Goran Gajski, Katarina Matković, Luka Delić, Marko Gerić

**Affiliations:** Mutagenesis Unit, Institute for Medical Research and Occupational Health, 10000 Zagreb, Croatia

**Keywords:** vegetarians, pescatarians, omnivores, DNA damage, genome instability, comet assay, human biomonitoring, health effects

## Abstract

DNA damage is known to be associated with many adverse health outcomes, including cancer and chronic diseases, but also with the process of aging. Empirical evidence has shown that environmental exposures, such as certain lifestyle factors, can affect a variety of health-related biomarkers and also impact the stability of DNA through the upregulation of the antioxidant defense system and alteration of its repair capacity. In addition to exercising, diet is an important lifestyle factor that can affect the development of a variety of chronic diseases and growing evidence suggests that plant-based diets, including vegetarianism, may promote health, longevity, and well-being. Therefore, we aimed to assess the primary DNA damage in 32 young healthy females from Zagreb, Croatia, based on their dietary preferences. The participants were divided into two groups: vegetarians and non-vegetarians, where the non-vegetarian group was further divided into omnivores (traditional mixed diet) and pescatarians (consumption of fish and seafood). According to statistical analysis, the DNA damage measured in whole blood cells expressed as the % tail DNA was significantly (*p* < 0.05) higher in vegetarians (3.6 ± 1.1%) compared to non-vegetarians (2.8 ± 1.0%). When further dividing the participants into specific sub-groups, lower DNA damage was observed amongst omnivorous subjects (3.2 ± 0.8%) compared to vegetarians, with the lowest DNA damage found in females practicing a pescatarian diet (2.4 ± 1.1%). Although a vegetarian diet can lead to a higher intake of specific vitamins and micronutrients, it can also lead to a deficiency of iron, calcium, and total proteins, which may affect genome stability and induce oxidative stress. Even though our results have shown that the pescatarian diet would be more beneficial in terms of maintaining DNA integrity, further research should be carried out to assess how specific dietary preferences affect DNA integrity on a larger scale.

## 1. Introduction

Damage to DNA molecules is recognized as one of the initial steps in the development of chronic diseases, cancer, and the aging process [1,2,3]. Increasing evidence indicates that dietary factors and exercise may impact various health-related biomarkers and can also affect the stability of DNA molecules by upregulating the antioxidant defense system and modulating repair capacity [4,5]. Numerous bioactive compounds detected in plant foods have the potential to reduce oxidative stress and inflammation as well as protecting against DNA damage through different modes of action, such as induction of DNA repair or by increasing resistance to oxidative damage [6,7]. In many clinical trials, foods and beverages rich in antioxidants have shown the ability to reduce DNA damage and increase DNA repair or antioxidant capacity [8,9,10,11]. This indicates that diet itself can be used as a modulator of different cell processes, especially in individuals with molecular signatures that can create excess DNA damage, oxidative stress, as well as inflammation.

Previously, we reported variances in different health-related biomarkers among people of both sexes practicing either traditional mixed or vegetarian diets. Our results showed that vegetarians have a lower nutritional status of some nutrients such as vitamins B_12_ and D as well as Zn, Ca, and Cu. This was accompanied by decreased antioxidant defenses in terms of glutathione as well as increased homocysteine levels. Moreover, vegetarians displayed increased DNA damage in the form of micronuclei and strand breaks as well as shorter telomeres. These findings suggest that the supplementation of animal-derived nutrients to this specific dietary group could lead to beneficial effects in terms of certain health-related biomarkers. On the contrary, our results also revealed that non-vegetarians displayed a higher level of some toxic metals, such as As and Hg [12]. Moreover, we also revealed that vegetarianism resulted in improved pancreatic β-cell function in women. The favorable adiponectin and insulin sensitivity responses in females reveal a distinct outcome of diet-to-metabolic homeostasis, demonstrating an interesting pattern of sexual dimorphism concerning the positive metabolic effect of vegetarianism [13].

Therefore, in this study, we wanted to evaluate primary DNA damage in young healthy female volunteers based on their dietary preferences. The subjects were divided into vegetarians (lacto–ovo vegetarians) and non-vegetarians, with the non-vegetarian group being further divided into omnivores (traditional mixed diet) and pescatarians (consumption of fish and seafood). The comet assay was used to evaluate the level of primary DNA damage in the female subjects. The assay itself is a well-known and accepted biomonitoring instrument used for the assessment of the effects of different lifestyle, environmental, occupational, and even dietary exposures on the levels of DNA damage in various human cells [14,15,16]. The comet assay is capable of recognizing DNA damage resulting from single- and double-strand breaks, single-strand breaks related to incomplete excision repair sites, DNA cross-links, as well as alkali-labile sites [17,18]. Furthermore, the measurement of DNA damage with this method could serve as a tool to predict the likelihood of developing any form of cancer and the associated risk of mortality. Such epidemiological evidence can emphasize the importance of incorporating the comet assay as a preventative measure for various non-communicable diseases [19,20].

## 2. Materials and Methods

### 2.1. Study Sample and Participant Selection

This study was conducted on a group of young female subjects (*n* = 32). All recruited subjects were from the general Zagreb (Croatia) population. At the time of the interview and blood sampling, all subjects were in a healthy condition and were free from any acute illnesses. Prior to donating blood for the study, all participants were occupationally not exposed to any hazards, including diagnostic ionizing radiation, and had not taken any medication such as antibiotics and/or steroid therapy for at least one year. All participants provided their written informed consent and completed a questionnaire intended to obtain demographic data, health status, and lifestyle factors such as smoking habits and alcohol consumption. Moreover, they gave information regarding their family history of cancer as well as data on previous or existing exposure to medication and diagnostic radiation that could interfere with the comet assay results. The study was approved by the Ethics Committee of the Institute for Medical Research and Occupational Health, Zagreb (Croatia) with ensured data privacy.

### 2.2. Blood Sampling

A trained medical professional collected venous blood into sterile heparinized tubes (Becton Dickinson, Franklin Lakes, NJ, USA) in the morning hours (between 7 and 10 a.m.). Subsequently, we handled their blood samples uniformly, by assigning random codes, storing them at 4 °C in light-protected conditions, and processing them as soon as possible (within 3 h after the sampling).

### 2.3. Alkaline Comet Assay

#### 2.3.1. Procedure

The alkaline comet assay was performed based on the protocol by Collins et al. [17] following MIRCA guidelines [21]. Whole-blood samples (5 µL) were embedded in an agarose sandwich gel in the 0.5% low melting point (LMP) agarose layer (Sigma, St. Louis, MO, USA) on fully frosted slides. Afterward, they were placed in freshly made cold lysis solution (1% Triton X-100 (Sigma, St. Louis, MO, USA), 1% sodium N-lauroyl sarcosinate (Sigma, St. Louis, MO, USA), 2.5 M NaCl (Kemika, Zagreb, Croatia), 10% dimethyl sulfoxide (Kemika, Zagreb, Croatia), 10 mM Tris−HCl (Sigma, St. Louis, MO, USA; Kemika, Zagreb, Croatia), and 100 mM disodium EDTA (Sigma, St. Louis, MO, USA), pH 10), and left in the refrigerator (at 4 °C) during the night. After the cells were lysed and the DNA was unfolded, the slides were placed in electrophoresis solution (1 mM disodium EDTA (Sigma, St. Louis, MO, USA), 300 mM NaOH (Kemika, Zagreb, Croatia), pH 13) for 20 min (at 4 °C) and then they were electrophoresed for another 20 min (at 1 V/cm). Subsequently, the slides were washed with 0.4 M Tris−HCl buffer (pH 7.5) and dyed with ethidium bromide (10 μg/mL; Sigma, St. Louis, MO, USA) before the inspection.

#### 2.3.2. Image Analysis

We created two slides for each participant and randomly evaluated a total of 100 comets per slide (200 comets in total). Selected comets were analyzed using an epifluorescence microscope (Olympus BX-51, Tokyo, Japan) armed with suitable filters as well as the Comet Assay IV (Perceptive Instruments Ltd., Haverhill, Suffolk, UK) software intended to analyze them. Tail intensity (i.e., % tail DNA) was used as the preferred comet assay descriptor which is expressed as % of total DNA fluorescence in the tail of the comet.

### 2.4. Statistical Analysis

Statistical analysis was performed using R statistical programming language version 4.1.2 and TIBCO Statistica™ (Palo Alto, CA, USA). Elementary statistical parameters were obtained using descriptive statistics. We tested the normality of the data with Shapiro–Wilk’s W test. The data were then log-transformed. The homogeneity of variance for each variable was tested with Bartlett’s Test. The differences in % tail DNA between the three tested groups (omnivorous, vegetarians, and pescatarians) were tested using ANOVA (type III) and the Scheffe test. The differences in % tail DNA between vegetarians and non-vegetarians were tested using a *t*-test with Bonferroni correction for *p*-value. The significance level for all tests was set at *p* < 0.05.

## 3. Results

### 3.1. Population Characteristics

This study encompassed a group of young female non-smoking volunteers (*n* = 32) aged between 25 and 35 years (the average age was 31.2 years and their median age was 30 years) with comparable socioeconomic statuses. They were divided into several dietary groups: vegetarian (*n* = 16) and non-vegetarian (*n* = 16) dietary groups. Vegetarian participants were practicing a lacto–ovo vegetarian diet which includes eggs and dairy as products of animal origin but excludes meat, poultry, fish, and seafood. The mean number of years practicing a vegetarian diet was 10.7 years. Moreover, the non-vegetarian group was divided into two subgroups, namely, omnivores (*n* = 8) and pescatarians (*n* = 8). Detailed population and lifestyle characteristics and their comet assay values are presented in Table 1.

All participants were selected from the general Croatian population and lived in the same city of Zagreb (the capital of Croatia). Female volunteers displayed comparable patterns of physical activity (regular exercise at least two times per week for at least half an hour) and comparable education level (high school and university). The participants in our study had no history of smoking, exposure to ionizing radiation, steroid therapy, or antibiotics for at least one year prior to blood donation. In addition, they were free of any occupational exposures that might have hindered the results of our study and had no known chronic illnesses or inherited genetic disorders. They were all in the normal weight range with their body mass index (BMI) above 18.5 and below 25 kg/m^2^.

### 3.2. Dietary Preferences and Genome Damage

**Figure 1:**
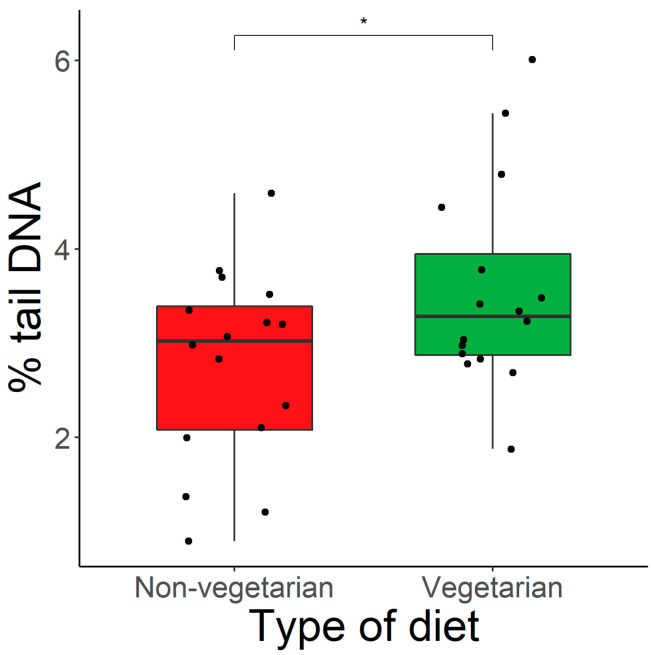
The difference in % tail DNA among vegetarians and non-vegetarians; * *p* < 0.05.

**Figure 2:**
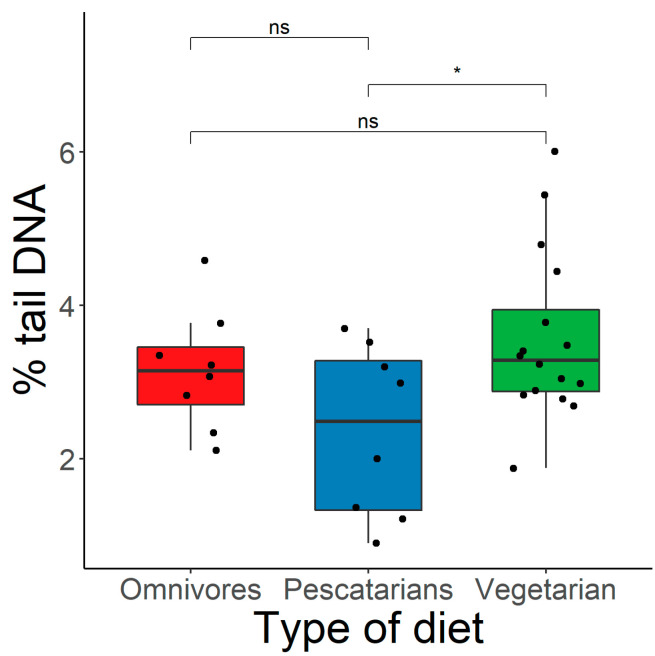
The differences in % tail DNA among three tested groups (omnivorous, vegetarians, and pescatarians); ns—non-significant, * *p* < 0.05.

According to our results (presented as box plots), damage to DNA molecules measured in whole blood cells, as evaluated by the alkaline comet assay and expressed as the % of tail DNA, was significantly (*p* < 0.05) higher in vegetarian subjects (3.6 ± 1.1%) compared to non-vegetarian ones (2.8 ± 1.0%; Figure 1). Moreover, when divided into specific sub-groups, we observed lower DNA damage in omnivorous subjects (3.2 ± 0.8%) compared to vegetarians with the lowest DNA damage observed in females practicing a pescatarian diet (2.4 ± 1.1%). It has to be pointed out that the only statistically significant (*p* < 0.05) difference was detected between vegetarians and pescatarians (Figure 2).

## 4. Discussion

Diet is regarded as one of the most important environmental contributors that can influence the occurrence of a variety of chronic illnesses [22,23] and there is increasing evidence that plant-based diets including vegetarianism may promote health, longevity, and well-being. Moreover, it is postulated that vegetarian and vegan diets and their specific components can lower the rate of different chronic disorders including diabetes, cancer, and cardiovascular disease [4,24,25,26]. Today we have many different types of vegetarian diets that are based on the level to which one is avoiding the consumption of animal products [27]. This can subsequently influence micronutrient intake; however, there are also differences in omnivorous diets regarding the quantity and the type of meat that is preferentially consumed. Although a vegetarian diet may result in a higher intake of specific micronutrients, as well as vitamins that may provide antioxidant defenses, there is also a possibility of a deficiency in some other micronutrients that are involved in DNA metabolism and its stability [4,12]. In line with the above-mentioned studies, this particular study aimed to investigate whether there is a possible difference in the level of DNA damage among young women practicing different diets, namely vegetarian and non-vegetarian ones.

To date, several studies have evaluated the impact of the vegetarian diet on genome integrity with non-conclusive results (reviewed in Gajski et al. [4]). Vegetarian Seventh-Day Adventists displayed lower chromosomal damage, expressed as the frequency of sister chromatid exchanges, in comparison with the general public. Still, inside the group of Seventh-Day Adventists, the authors did not observe differences among vegetarians and omnivores, suggesting that the overall lifestyle could be the cause for such differences [28]. Studies from Slovakia showed a higher amount of oxidative DNA damage, particularly concerning elderly omnivores but failed to observe differences in total DNA strand breaks, chromosome aberrations, or micronuclei frequency (an indication of chromosome breakage [29,30,31,32,33]) levels between different dietary groups [34,35,36]. In the papers by Kotova et al. [37] and Dhavan et al. [38] from Swedish and Indian populations, respectively, vegetarians had a lower baseline cytogenetic status and a reduced amount of DNA strand breaks compared to omnivore subjects.

It has to be pointed out that there are studies that failed to find differences between eating preferences and genome damage, such as another study from India showing no differences in DNA damage between vegetarian versus omnivore groups [39]. No differences were found regarding DNA damage and micronuclei frequency among subjects practicing uncooked veganism and omnivores [40] as well as the difference in micronucleated cells among vegetarians and meat eaters in research done by Davies et al. [41]. Concerning DNA damage and age, a study done by Fenech and Rinaldi [42] showed that young males practicing a vegetarian diet had a higher micronuclei frequency and middle-aged males practicing vegetarianism had a lower micronuclei frequency whereas, in older male and female subjects practicing a vegetarian diet, there was no difference in micronuclei compared to omnivores. Our previous results [12] on both males and females showed significantly higher DNA strand breaks and micronuclei frequencies in vegetarians compared to matched non-vegetarians, together with the several other blood and plasma biomarkers tested.

Generally speaking, vegetarians could have a higher intake of certain specific micronutrients as well as vitamins that are necessary for the proper functioning of the antioxidant defense system. On the other hand, they could be experiencing a deficiency in other micronutrients with a role in DNA metabolism and stability [4]. For instance, dietary antioxidants are involved in the cellular antioxidant defense system, which could strengthen the protection of DNA molecules by modulating several signaling pathways and gene expression, protecting and repairing DNA damage, and increasing the free radical scavenging capacity that arises through metabolic reactions [43,44]. In addition, it has been shown that higher consumption of antioxidant-rich vegetables and/or fruits can lower levels of oxidatively damaged DNA [45,46,47]. Since DNA damage is regarded as the initiating event in carcinogenesis, eating larger quantities of vegetables and fruits could be helpful in cancer protection in line with the prevention of free radical attacks on macromolecules [48,49], although there are carnivorous diets rich in fruits and vegetables as well as different plant-based types of diets which do not necessarily completely avoid meat and other animal products, such as the Mediterranean type of diet [50]. Moreover, antioxidants found in plants, such as flavonoids, carotenoids, and certain vitamins (C and E), can help protect from against various diseases by lowering the level of oxidative damage [51]. A certain downfall of avoiding meat consumption is that meats and dairy products are sources of vitamins belonging to the B group that are vital for cell division and erythropoiesis [52,53] as well as for DNA metabolism and stability [48]. Moreover, vitamin D that exists mostly in animal food sources is also beneficial in maintaining DNA integrity. Its role is primarily connected with DNA damage prevention but it also regulates the growth rate of cells [54]. Additionally, vegetarians tend to lack iron, calcium, and total proteins which may lead to genome instability as well as oxidative stress [55,56]. Hence, minimizing or eliminating animal foods from one’s diet may lead to the accumulation of unrepaired DNA damage that can initiate genome instability and possibly induction of cancer [57] and its related costs [58].

We also have to acknowledge several limitations of the study. This study was cross-sectional in its design; hence, causation could not be established. As in many other studies, the number of participants was limited; however, such studies are valuable to use in future meta-analyses where the statistical power of the results would be higher. The volunteers self-reported their dietary preferences, which leaves room for the potential of underestimating or overestimating the intake of certain food items, as well as confusion about the classification of certain dietary categories.

## 5. Conclusions

In summary, a number of studies failed to associate specific dietary preferences with DNA damage. Nevertheless, conflicting reports mentioned above indicate that there could be some differences regarding dietary preferences and measured genome instability biomarkers that could also be dependent on the country of origin and/or urban and rural differences in dietary intake. Based on our results, it seems that a pescatarian diet, which could be defined as plant-based if it contains an adequate intake of fish and seafood, could be a more beneficial diet in terms of maintaining DNA integrity. Although toxic metals such as mercury, increasing microplastics, as well as other persistent toxins found in fish could increase our risk for DNA damage and other side effects, a considerable body of evidence including our study still suggests that the benefits of fish consumption may outweigh the risks. Moreover, our results indicate the necessity of conducting further research on a larger scale to investigate how specific dietary preferences may impact DNA integrity and consequently our health to contribute to the everlasting discussion that started more than 130 years ago [59,60].

## Figures and Tables

**Table 1:** Population characteristics in vegetarians versus non-vegetarians (*n* = 32) and their related sub-groups.

		Vegetarians (*n* = 16)	Non-Vegetarians (*n* = 16)		
				Omnivores (*n* = 8)	Pescatarians (*n* = 8)
Age (years)					
	Mean ± SD	31.4 ± 3.1	31.0 ± 3.5	31.0 ± 3.9	31.0 ± 3.27
	Range	25–35	26–35	26–35	26–35
Vegetarianism (years)					
	Mean ± SD	10.7 ± 3.8	-	-	-
	Range	4–18	-	-	-
Tail intensity (% tail DNA)					
	Mean ± SD	3.6 ± 1.1 *^,#^	2.8 ± 1.0	3.2 ± 0.8	2.4 ± 1.1
	Range	1.9–6.0	0.9–4.6	2.1–4.6	0.9–3.7

* Statistically significant increase compared to non-vegetarians (*p* < 0.05); ^#^ statistically significant increase compared to pescatarians (*p* < 0.05).

## Data Availability

The original contributions generated for this study are included in the article. Further inquiries can be directed to the corresponding author upon reasonable request.
